# Carvacrol reduces adipogenic differentiation by modulating autophagy and ChREBP expression

**DOI:** 10.1371/journal.pone.0206894

**Published:** 2018-11-12

**Authors:** Sonia Spalletta, Vincenzo Flati, Elena Toniato, Jacopo Di Gregorio, Antonio Marino, Laura Pierdomenico, Marco Marchisio, Gabriella D’Orazi, Ivana Cacciatore, Iole Robuffo

**Affiliations:** 1 Institute of Molecular Genetics, National Research Council, Section of Chieti, Chieti, Italy; 2 Department of Medical, Oral and Biotechnological Sciences, University of Chieti-Pescara, Chieti, Italy; 3 Department of Biotechnological and Applied Clinical Sciences, University of L’Aquila, L’Aquila, Italy; 4 Department of Medicine and Aging Science, University “G. d’Annunzio”, Chieti-Pescara, Chieti, Italy; 5 Aging Research Center (CeSI-Met), Gabriele D’Annunzio University Foundation, Chieti, Italy; 6 Department of Pharmacy, University “G. d’Annunzio” Chieti-Pescara, Chieti, Italy; Univerzitet u Beogradu, SERBIA

## Abstract

**Objective:**

Obesity is the result of white adipose tissue accumulation where excess of food energy is stored to form triglycerides. De novo lipogenesis (DNL) is the continuous process of new fat production and is driven by the transcription factor ChREBP. During adipogenesis, white adipocytes change their morphology and the entire cell volume is occupied by one large lipid droplet. Recent studies have implicated an essential role of autophagy in adipogenic differentiation, cytoplasmic remodelling and mitochondria reorganization. The phenolic monoterpenoid carvacrol (2-methyl-5-[1-methylethyl]phenol), produced by numerous aromatic plants, has been shown to reduce lipid accumulation in murine 3T3-L1 cells during adipogenic differentiation by modulating genes associated with adipogenesis and inflammation. Therefore, the aim of this study was to evaluate whether carvacrol could affect autophagy and ChREBP expression during adipogenic differentiation.

**Methods:**

The study was carried on by using the murine 3T3-L1 and the human WJ-MSCs (Wharton’s jelly-derived mesenchymal stem cells) cell lines. Cells undergoing adipogenic differentiation were untreated or treated with carvacrol. Adipogenic differentiation was assessed by analyzing cellular lipid accumulation with Oil-Red O staining and by ultrastructural examination with TEM. Autophagy was evaluated by western immunoblotting of autophagy markers LC3B and p62/SQSTM and by ultrastructural examination of autophagic bodies. Autophagic flux was evaluated by using autophagy inhibitor cloroquine (CQ). ChREBP expression levels was assessed by both western blotting and immunoelectron microscopy and ChREBP activity by analysis of adipogenic target genes expression.

**Results:**

We found that carvacrol reduced adipogenic differentiation of about 40% and 30% in, respectively, 3T3-L1 and in WJ-MSCs cells. The effect of carvacrol on adipogenic differentiation correlated with both reduction of autophagy and reduction of ChREBP expression.

**Conclusion:**

The results support the notion that carvacrol, through its effect on autophagy (essential for adipocyte maturation) and on ChREBP activity, could be used as a valuable adjuvant to reduce adipogenic differentiation.

## Introduction

Adipogenesis is a regulated cellular differentiation process during which the preadipocytes are transformed into mature adipocytes [1]. Adipocytes are the main constituent of adipose tissue and play a very important role in the homeostatic control of whole body metabolism [2]. Their primary function is to control energy balance by storing triacylglycerol in periods of energy excess and by mobilizing it during energy deprivation. Adipocytes, other than storing fat, secrete several adipokines such as leptin [3,4] rendering adipose tissue the major endocrine organ with a significant impact on cell metabolism [5,6]. The overexpansion of adipose tissue mass plays a central role in obesity-related complications such as type-2 diabetes, hypertension, hyperlipidemia and atherosclerosis [7–9]. Furthermore, the proteins secreted by adipose tissue are also involved in the regulation of immune and neuroendocrine functions and in tumor progression [10–14]. The acute elevation of plasma free fatty acids (FFAs), caused by increased release from enlarged adipose tissue, activates the nuclear factor kappa-light-chain-enhancer of activated B cells (NF-κB) pro-inflammatory signaling in the liver and adipose tissues [15]. The result is the increased expression of several pro-inflammatory cytokines such as tumor necrosis factor-α (TNFα), interleukin (IL)-1β and IL-6 [16–18]. Liver and white adipose tissue are the principal organs involved in glucose and lipid homeostasis in mammals: thus, through *de novo* lipogenesis excess dietary carbohydrates are converted into triglycerides (TG). In this process, acetyl-CoA derived from glucose metabolism is used to synthesize fatty acids, which are subsequently esterified with glycerol to form TG. De novo lipogenesis process is driven by two major transcriptional regulators, the Sterol Response Element Binding Protein 1c (SREBP1c) [19] and the Carbohydrate Response Element Binding Protein (ChREBP) [20]. Both pathways are activated by increased insulin signalling in response to high glucose level [21, 22]. ChREBP specifically binds the carbohydrate response element (Chre) of the lipogenic genes Acetyl CoA carboxylase, Fatty acid synthase and Pyruvate kinase (LPK) [23]. ChREBP is expressed in all tissues although mainly in lipogenic organs as liver, adipose tissue and pancreas; it is activated in response to high concentrations of glucose [22] and can continuously move between the cytoplasm and nucleus independently of glucose concentrations [24]. The metabolite glucose-6-phosphate is necessary to mediate ChREBP activation [25].

Autophagy is a catabolic process through which the cell sequesters its intracellular material (i.e., unfolded proteins, protein aggregates, organelles, etc) for lysosomal degradation and recycling [26, 27]. The expression of microtubule associated protein light chain 3 (LC3), after conversion from LC3-I form to its lipidated autophagosome membrane-associated, activated LC3-II form, is considered a marker of autophagy along with the reduction of p62/SQSTM1 expression levels [28]. However, changes in p62/SQSTM1 levels could be cell type specific. Autophagy is an important mechanism for the maintenance of cellular homeostasis in energetic stress conditions such as energy deficiency and fasting but also in the presence of damaged proteins, irradiation and infection by microorganisms [29]. Moreover, autophagy has been shown to regulate adipose mass and differentiation [30–35]. Inhibition of autophagy in pre-adipocytes reduces both the accumulation of triglycerides and the expression of transcription factors involved in adipocyte differentiation. Therefore, a comprehensive investigation into the molecular mechanisms underlying adipogenesis and the way to modulate them, is of clinical relevance for the development of novel therapeutic treatments for obesity and associated metabolic syndromes.

**Fig 1:**
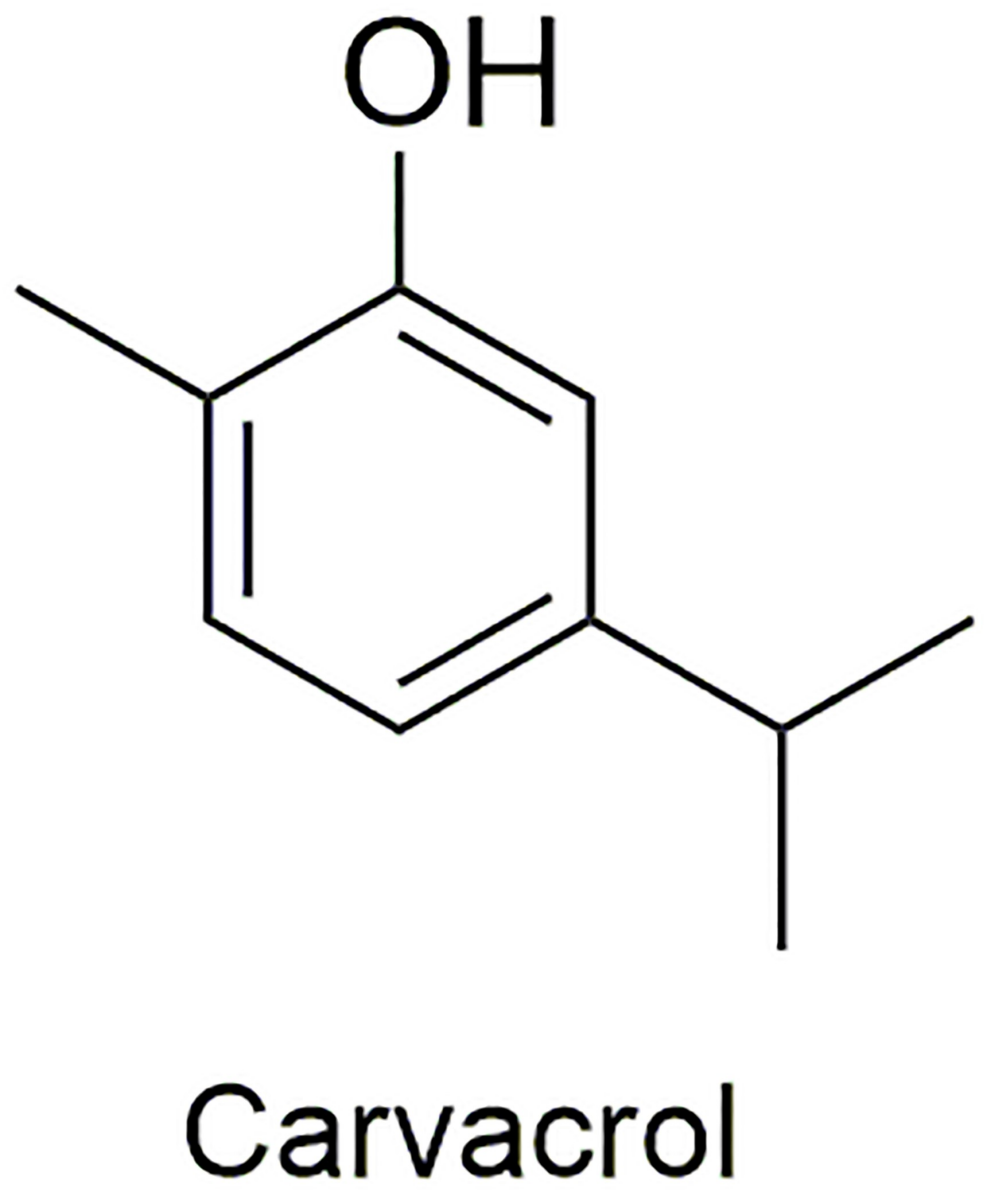
Chemical structure of carvacrol.

The phenolic monoterpenoid Carvacrol (2-methyl-5-[1-methylethyl]phenol) (Fig 1) is the main product of numerous aromatic plants including Origanum, Thymus, Satureja, Thymbra, Coridothymus and Lippia [36].

Several reports indicate that carvacrol exhibits different activities such as antimicrobial [37,38], fungicidal [39], anti-inflammatory [40], analgesic [40], antioxidant [41,42], anti-proliferative [43], anti-carcinogenic and antiplatelet [44]. Moreover, this natural compound promotes cancer cell death via necrosis and/or apoptosis [43]. Carvacrol has been shown to reduce lipid accumulation in murine 3T3-L1 cells during adipogenic differentiation by modulating genes associated with adipogenesis and inflammation [45,46].

In this study we aimed to evaluate the effect of carvacrol on adipogenic differentiation in human WJ-MSCs (Whartons jelly’s derived mesenchymal stem cells) cells compared to murine 3T3-L1 cells. We found that carvacrol reduced adipogenic differentiation in both murine 3T3-L1 and WJ-MSCs cells along with reduction of autophagy (essential for adipocyte maturation) and of ChREBP activity. These findings make carvacrol an interesting novel anti-obesity phytochemical.

## Materials and methods

### Chemicals and reagents

Carvacrol (MW 150.22 g/mol, 98% purity) was obtained from Sigma, dissolved in dimethyl sulfoxide (DMSO) (Sigma-Aldrich) to produce a 10 mM stock solution and stored at -70°C before use; it was diluted into culture medium at different concentrations (from 10 to 50 μM) for the indicated times.

The inhibitor of autophagic protein degradation cloroquine (CQ) [28] (Sigma) was dissolved in dH_2_O and used at 25 μM for the indicated time.

### Cell Isolation and Cell Culture

#### 3T3-L1

Mouse embryo 3T3-L1 cells, obtained from ATCC (Manassas, VA, USA) were cultured in Dulbecco’s modified Eagle’s medium (D-MEM) (low glucose, 1 g/L) (Lonza) supplemented with 10% fetal bovine serum (FBS), 0.584 g/L glutamine, 10 μg/ml penicillin, 10 μg/ml streptomycin, at 37°C in 5% CO2.

#### WJ-MSC(Whartons jelly’s derived mesenchymal stem cells)

With the consent of the parents, fresh human umbilical cords were obtained from full-term births; they were aseptically stored in sterile saline solution and processed within 6 hours from partum to obtain umbilical cord mesenchymal stem cells. After the removal of blood vessels, the abundant extracellular matrix of Wharton’s jelly was scraped off by a scalpel, finely cutted and centrifuged at 250 x G for 5 min at room temperature (RT); cell pellets were washed with serum-free (D-MEM) (Lonza). Next, cells were treated with collagenase type IV (2 mg/ml) (Sigma) for 16 hours at 37°C, washed in PBS and treated with 2.5% trypsin-EDTA (GIBCO) for 30 minutes at 37 °C under agitation. Finally, cells obtained from two subculture, namely WJ1 and WJ2, were washed in PBS and seeded in complete growth medium (HMSCGM) [47] with growth supplements (all from Lonza) in 5% CO2 at 37 °C. Cells were cultured exchanging the medium every 3–4 days until reaching confluence. Adherent cells were detached with 0.05% trypsin-EDTA, counted by Trypan Blue exclusion test, and re-seeded at 3000 cells/cm2 to reach 90% confluence. Cell culture institutional review board approval was obtained for all procedures by Chieti Hospital Istitutional Commettee on Human Experimentation N. 1879/09COET. This approval relates to the authorization to collect the umbilical cord for obtaining the Wharton’s jelly mesenchymal stem cells (WJ-MSCs), to be used exclusively for in vitro research.

### Fluorescence-activated cell sorting (FACS) flow cytometry

WJ-MSCs cells were examined for specific surface and intracellular expression markers by using flow cytometry. The following antibodies were used: fluorescein isothiocyanate-conjugated (FITC) anti-CD44 (CD44-FITC), anti-CD45 (CD45-FITC), anti-CD105 (CD105-FITC), phycoerythrin-conjugated (PE) anti-CD29 (CD29-PE) (all from Ancell (MN, USA); anti-CD90 (CD90-FITC) and anti-SSEA4 (SSEA4-FITC), anti-CD73 (CD73-PE) anti-CD34 (CD34-PE), anti HLA-DR (HLA-DR-PE) and anti-OCT3/4 (OCT3/4-PE), Alexa488 anti HLA-ABC and conjugated anti-Sox2 (Sox2-Alexa488) (all from Becton Dickinson (BD, San Jose, CA).

Adherent cells at 80% confluence were treated with EDTA at 37°C for 10 min. Cells were washed with 2 ml Phosphate Buffered Saline (PBS, 0.1% sodium azide and 0.4% bovine serum albumin) and centrifuged at 400g for 8 min at 4°C. For staining of surface antigens, samples were resuspended in 100 μl washing buffer containing appropriate surface antibody and incubated for 30 min at 4°C in the dark. Cells were washed with 3 ml of washing buffer, centrifuged at 400 x G for 8 min at 4 °C and incubated with 1ml 0.5% paraformaldehyde for 5 min at RT; finally, cells were washed in PBS, centrifuged (400 x G for 8min at 4°C) and stored at 4 °C in the dark until acquisition.

For staining of intracellular antigens, cells were resuspended in 1 ml of FACS lysing solution (BD), vortexed and incubated for 10 min at room temperature (RT) in the dark. Samples were centrifuged and incubated at RT for 10 min in the dark in 1ml of Perm2 (BD). After washing cells were resuspended in 100 μl washing buffer containing appropriate intracellular antibody and incubated for 30 min at 4°C in the dark. Cells were then washed, centrifuged and resuspended in 0.5% paraformaldehyde for 5 min at room temperature and stored at 4°C in the dark until acquisition. Cells were analysed on a FACSCanto II flow cytometer (BD) using DIVA software (BD). Twenty thousand non-debris events in the morphological gate were recorded for each sample. Data were analysed using FlowJo software (Treestar, Ashland, OR). Mean Fluorence Intensity Ratio (MFI ratio) was calculated dividing the MFI of positive events by the MFI of negative events.

### Adipogenic differentiation

#### 3T3-L1

Mouse embryo 3T3-L1 cells were seeded at a density of 6x10^4^ per well in 6-well plates. After reaching confluence (8.8x10^6^/cm^2^), cells were induced to differentiate in adipogenic medium (high glucose D-MEM supplemented with 10% FBS, 1.7 μmol /L insulin, 0.5 mM 3-isobutyl-1-methylxanthine (IBMX), 1μM dexamethasone, 10 μg/ml penicillin, 10 μg/ml streptomycin) for at least seven days, without changing the culture medium but only adding fresh medium to avoid losing the viable differentiated but detached cells. Cells were processed after 7 days of treatment.

#### WJ-MSC

For adipogenic differentiation, WJ-MSC cells were plated in 6-well plates at a density of 2X 10^4^/cm^2^ in (DMEM) supplemented with 10% FBS, 0.584g/L glutamine, 10 μg/ml penicillin, 10 μg/ml streptomycin, at 37°C in 5% CO_2_. When cells reached confluence, culture medium was replaced with adipogenic medium (as for 3T3-L1). The adipogenic differentiation was achieved after 17 days treatment by supplementing differentiation medium with only insulin every 2–3 days.

### Oil-Red O staining to detect adipogenic differentiation

Lipid accumulation in mature adipocytes, undergoing differentiation with or without carvacrol, was determined by Oil-Rd O staining (Sigma) in 3T3-L1 at 7 days and in WJ-MSC at 17 days of differentiation. Cells grown on 6-wells were fixed in 4% formaldehyde in PBS for 10 min, washed with 60% isopropanol and stained with 0.2% Oil-Red O in 60% isopropanol for 10 min. To remove unincorporated dye, cells were washed several times with water and de-stained in 60% isopropanol for 15 minutes. Cell acquiring red staining were counted in four adjacent 1 mm squares were counted, blind to group, with an inverted microscope and using criterion described for quantification of adipocyte differentiation [48]. Cells were then observed at optical Zeiss microscope and photographed. The percentage of cells that underwent adipogenic differentiation was expressed as number of cells Red- Oil positive/total.

Lipid quantification was obtained by dissolving stained cellular oil droplets in 60% isopropanol and quantified by spectrophotometrical analysis at 580 nm. The supernatant containing detached cells, negative for Trypan blue staining (viable), was collected after 7 days (for 3T3-L1) and after 17 days (for WJ-MSC) of differentiation, centrifuged, stained with Oil-Red O and quantified by spectrophotometrical analysis. Oil-Red O was expressed as the summary of stained adherent and detached cells.

### Viability assay

For viability assay, confluent cells were plated in 6-wells dishes and treated with the indicated reagents, according to dose and time. Cells floating in the medium, detached during adipocytic differentiation, were collected and cell viability was determined by Trypan blue exclusion by direct counting with a haemocytometer. The viable cells (trypan blue negative) were collected after 7 days (for 3T3-L1) and 17 days (for WJ-MSC) of adipocyte differentiation, stained with Oil-Red Oil and lipid content quantified by spectrophotometrical analysis. The percentage of cell death, as blue/total cells, was assayed by scoring about 200 cells per well in triplicate.

### Western blotting

Protein extracts were run on precast Bis-Tris protein gels (Invitrogen, Thermo Fisher Scientific, Waltham USA) before transferring onto IBLOT PVDF Stacks (Invitrogen, Thermo Fisher Scientific, Waltham USA). The membranes were stained with Ponceau Red (Sigma-Aldrich, Milano, Italy) in order to verify the proper protein transfer, and then blocked at RT for 1 hour with 5% non-fat dry-milk in TBST containing 0.1% Tween20. The expression of different proteins was analyzed by Western blotting with antibodies diluted in 5% non-fat milk in TBST 0.1% Tween20, to the following: ChREBP (1:100), LC3B (1:1000) and p62 (1:1000) (Santa Cruz Biotechnology, Heidelberg, Germany and Cell Signaling, Leiden, Netherlands); all antibodies were incubated o/n at 4°C. The membranes were then washed 3 times for 10 minutes with TBST. Then, membranes were incubated for 1 hour at RT with anti-rabbit or anti-goat (depending on the primary antibody) HRP-conjugated secondary antibody (Santa Cruz Biotechnology, Heidelberg, Germany) diluted 1/2000 in TBST containing 5% non-fat milk. The membranes were washed 3 times for 10 minutes, incubated in SuperSignal West Pico (Thermo Fisher Scientific Inc, Pierce Biotechnology, Rockford, IL, USA) chemiluminescent substrate and detected using a ChemiDoc XRSplus imaging system (Bio-Rad Laboratories, Milano, Italy). The optical densities of blot bands were finally determined using a computer-assisted densitometer (ImageJ, U.S. National Institutes of Health, Bethesda, Maryland, USA), normalized with the ß-actin internal control.

### RNA extraction and semi-quantitative reverse transcription (RT)-PCR analysis

Cells were harvested in TRIzol Reagent and total RNA was isolated following the manufacturer’s instructions (Invitrogen). The first strand cDNA was synthesized from 2 μg of total RNA with MuLV reverse transcriptase kit (Applied Biosystems). Semi-quantitative Reverse-Transcribed (RT)-PCR was carried out by using Hot-Master Taq polymerase (Eppendorf) with 2 μl cDNA reaction and genes specific oligonucleotides under conditions of linear amplification, as previously reported [49]. PCR products were run on a 2% agarose gel and visualized with ethidium bromide. The housekeeping 28S gene, used as internal standard, was amplified from the same cDNA reaction mixture. Densitometric analysis was applied to quantify mRNA levels compared to control gene expression. Primer sequences are available upon request.

### Transmission electron microscopy (TEM)

For conventional TEM analysis, cells were fixed with 2.5% glutaraldehyde in 0.1 M cacodylate buffer, pH 7.4, postfixed with 1% OsO4, dehydrated in graded ethanol and embedded in Epon 812. Thin sections were then cut and stained with uranyl acetate and lead citrate for ultrastructural observation. Sections were photographed using a Philips 268 D electron microscope (FEI). Autophagic bodies were counted at 11000 magnification image x 100 cells.

### Immunoelectron-microscopy

For immunolocalization of ChREBP protein, cells were fixed with a mixture of 4% paraformaldehyde/1% glutaraldehyde in 0.1 M cacodylate buffer, pH 7.4, for 1 h and washed twice with the same buffer. Cells were dehydrated sequentially in 50, 75 and 90% dimethylformamide and embedded in Bioacryl resin (British Biocell, Cardiff, United Kingdom), followed by UV polymerization. Ultrathin sections were cut and mounted on 200-mesh nickel grids. To block nonspecific binding sites, grids were treated for 10 min at RT with a blocking buffer TBS (0.05 M Tris-Hcl, 1% sodium azide and 0.1% bovine serum albumin), pH 7.6. The grids were treated with 5% skim milk powder for 30 min and incubated overnight at 4°C with 1:30 dilution of anti-ChREBP (Santa Cruz biotechnology, inc). After several washes with TBS, grids were incubated for 1 h with 20 nm gold particles, conjugated with rabbit anti-goat IgG (Biocell, GB) diluted 1:10 in TBS, pH 8.2. Grids were then washed three times in TBS and once in distilled water, and stained with uranyl acetate. Controls were represented by untreated cells and by cells treated with secondary antibody alone. All sections were finally examined and photographed using a Philips 268D electron microscope (FEI). For each sample at least 100 cells were examined counting the total number of gold particles separately in cytoplasm and nuclei and assessing their presence per unit areas (10 cm^2^ at x11000) in both compartments.

### Statistical analysis

The data represent mean ±SD. All experiment unless indicated were performed at least three times. Comparisons between two groups were performed with an impaired Student’s *t* test. Differences of a *P*-values <0.05 were considered statistically significant.

## Results

### Cells isolated from Wharton’s jelly of human umbilical cord showed mesenchymal origin

**Fig 2:**
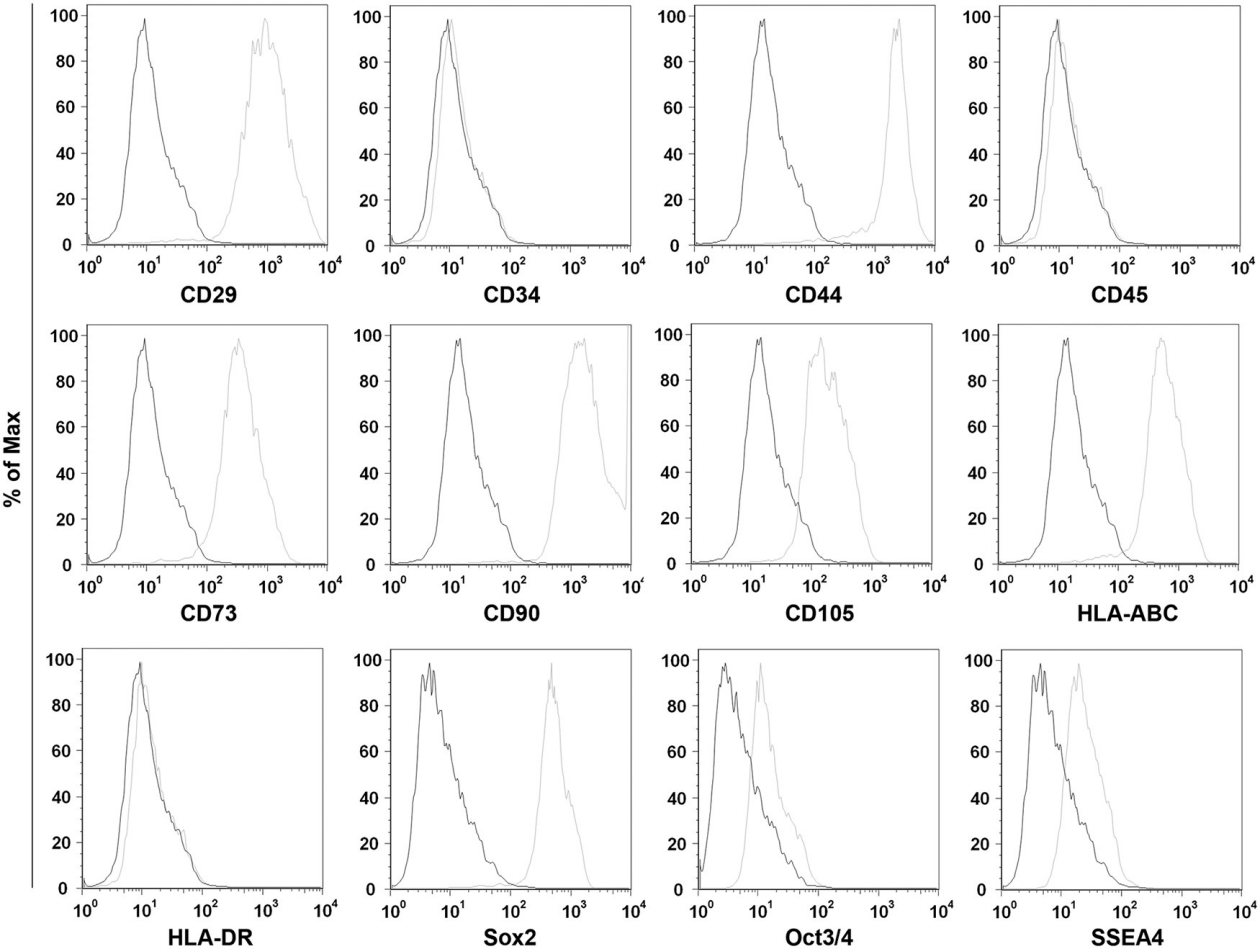
Cytofluorimetric analysis of the WJ cell culture. The histograms show the cytofluorimetric analysis of the WJ cell culture, surface and intracellular antigens expression profile: CD29, CD34, CD44, CD45, CD73, CD90, CD105, OCT3/4, SSEA4 and Sox2. Gray histograms represent cells stained with the expression markers; black histograms are the respective IgG isotype control.

Two subcultures of Wharton’ s jelly of human umbilical cord (WJ-MSC), WJ-1 and WJ-2, were subjected to flow cytometry analyse to evaluate their mesenchymal origin. Results show that both WJ-1 and WJ-2 cells were positive for some typical mesenchymal surface markers such as CD44, CD105, CD29, CD90 and CD73 (Table 1, Fig 2), while membrane markers that distinguish the hematopoietic progenitor cell line and vascular endothelial cells, such as CD34 and CD45, were not expressed (Table 1, Fig 2). In addition, cells were positive for both Oct3/4, a transcription factor involved in self-renewal of undifferentiated embryonic cell and necessary for pluripotency, and Sox2, a transcription factor that also plays a critical role for the maintenance of stem cell in the embryonic cells [47, 48, 50] (Table 1, Fig 2). Finally, cells were positive for HLA-ABC, also called major histocompatibility complex (MHC) class I, and for stage-specific embryonic antigen 4 (SSEA4), a stem cell marker while were negative for HLA-DR (MHC class II) (Table 1, Fig 2).

**Table 1:** Phenotype of WJ-MSC obtained from human umbilical cords.

Antigens	Phenotype	MFI Ratio ± SD
CD29	+++	80.3 ± 17.3
CD34	-	1.1 ± 0.1
CD44	+++	107.5 ± 21.2
CD45	-	1.2 ± 0.1
CD73	++	30.2 ± 5.3
CD90	+++	104.6 ± 22.3
CD105	++	10.3 ± 2.8
HLA-ABC	++	32.9 ±10.8
HLA-DR	-	1.3 ± 0.2
OCT3/4	+	3.8 ± 0.8
SSEA-4	+	4.6 ± 1.3
Sox-2	++	48.4 ± 9.3

- negative expression;

+ moderate expression;

++ positive;

+++ high expression.

Cut-off MFI Ratio Positivity>2.0

These results show that WJ-1 and WJ-2 subcultures were of mesenchymal origin.

### Carvacrol impairs adipogenic differentiation

**Fig 3:**
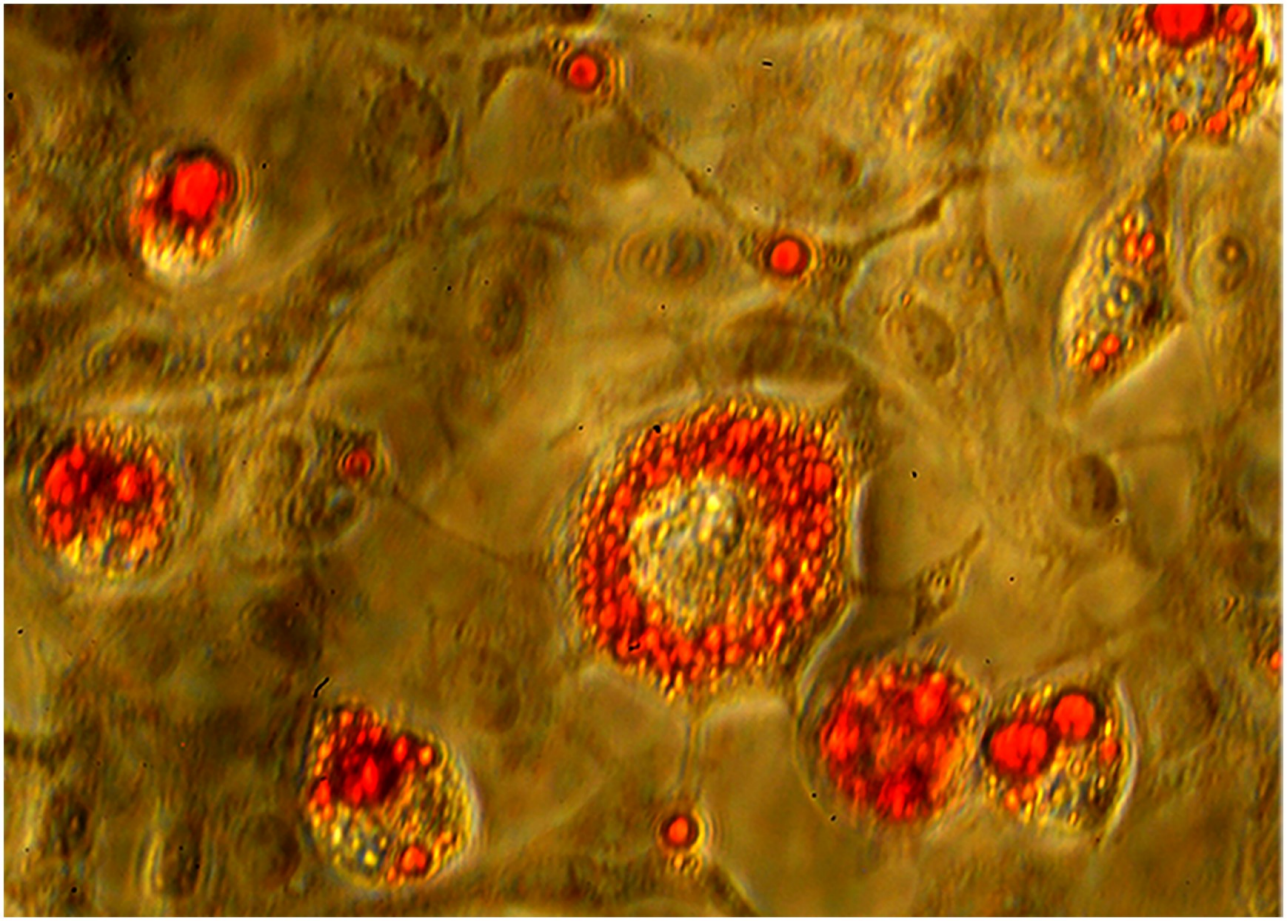
Oil Red Oil O staining. 3T3-L1 preadipocytes were differentiated in D-MEM (1-methyl 3-isobutylxanthine, dexamethasone, and insulin) medium for 7 days. Triglyceride accumulation visualized by Oil-Red O staining. Mature adipocytes show red large lipid vacuoles that occupied most of the cytoplasm. Original magnification: 400x.

Cell differentiation into adipocytes was evaluated by performing the Oil-Red O staining. Full differentiation was achieved after 7 days in 3T3-L1 cells, and after 17 days in WJ-MSCs cells. Fig 3 shows typical lipid accumulation in mature adipocytes with red large lipid vacuoles that occupied most of the cytoplasm.

**Fig 4:**
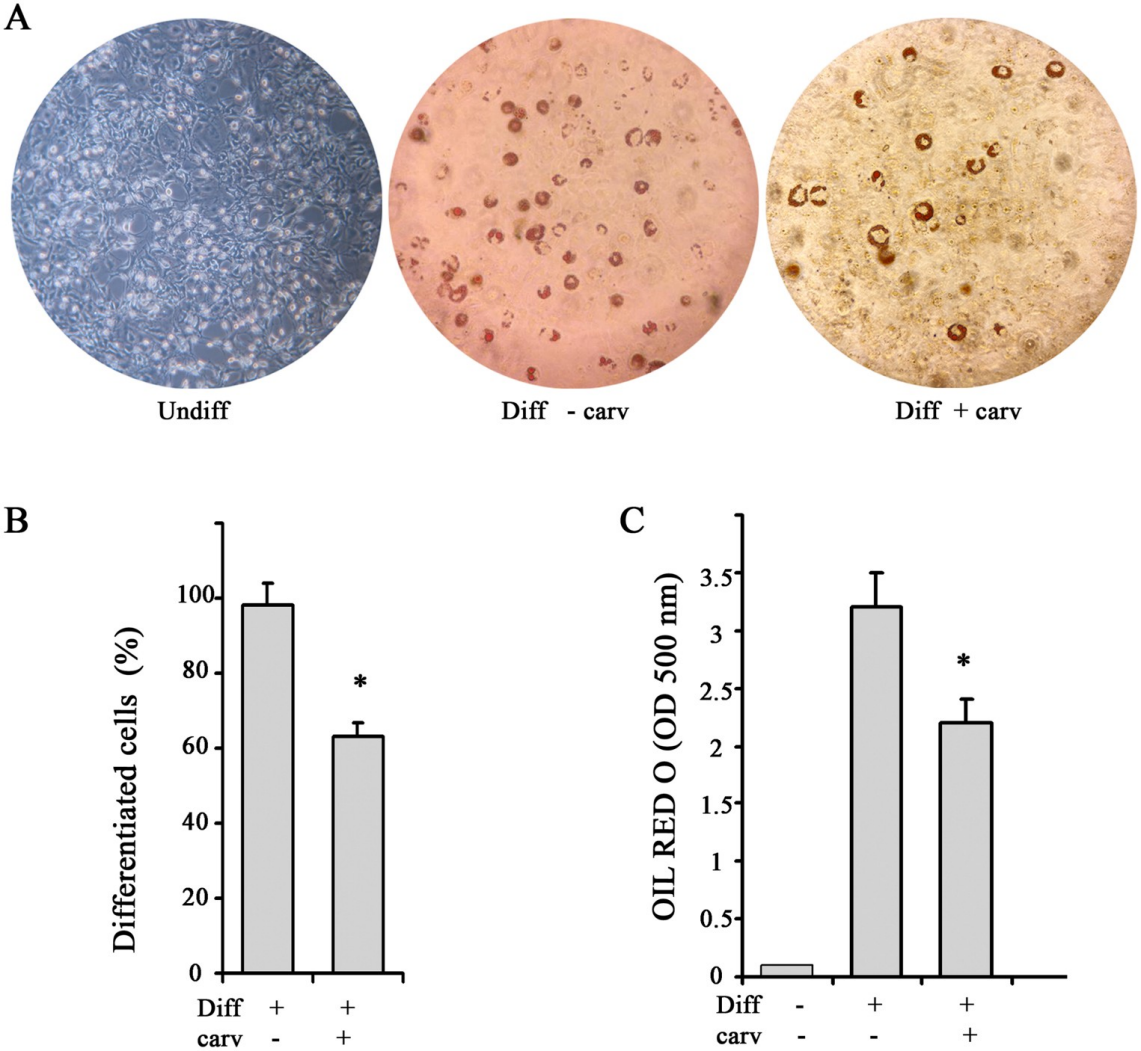
Reduction of adipocyte differentiation by carvacrol in 3T3-L1 cells. 3T3-L1 preadipocytes were grown in differentiation medium for 7 days (totally 90% differentiation) and treated with 25μ M carvacrol. After 7 days of adipogenic differentiation, visualization of triglyceride accumulation by Oil-Red O staining was conducted. (A) Cells were observed at optical Zeiss microscope and photographed. (B) Four adjacent 1 mm squares were counted, blind to group, using an inverted microscope and using criterion described for quantification of adipocyte differentiation [48]. The percentage of cells that underwent adipogenic differentiation was expressed as number of cells Red Oil positive/total. (C) Lipid accumulation was measured through a spectrophotometer. Data are presented as mean ± SD (*n 3)*. * p<0.05 (differentiated + carvacrol vs differentiated cells).

**Fig 5:**
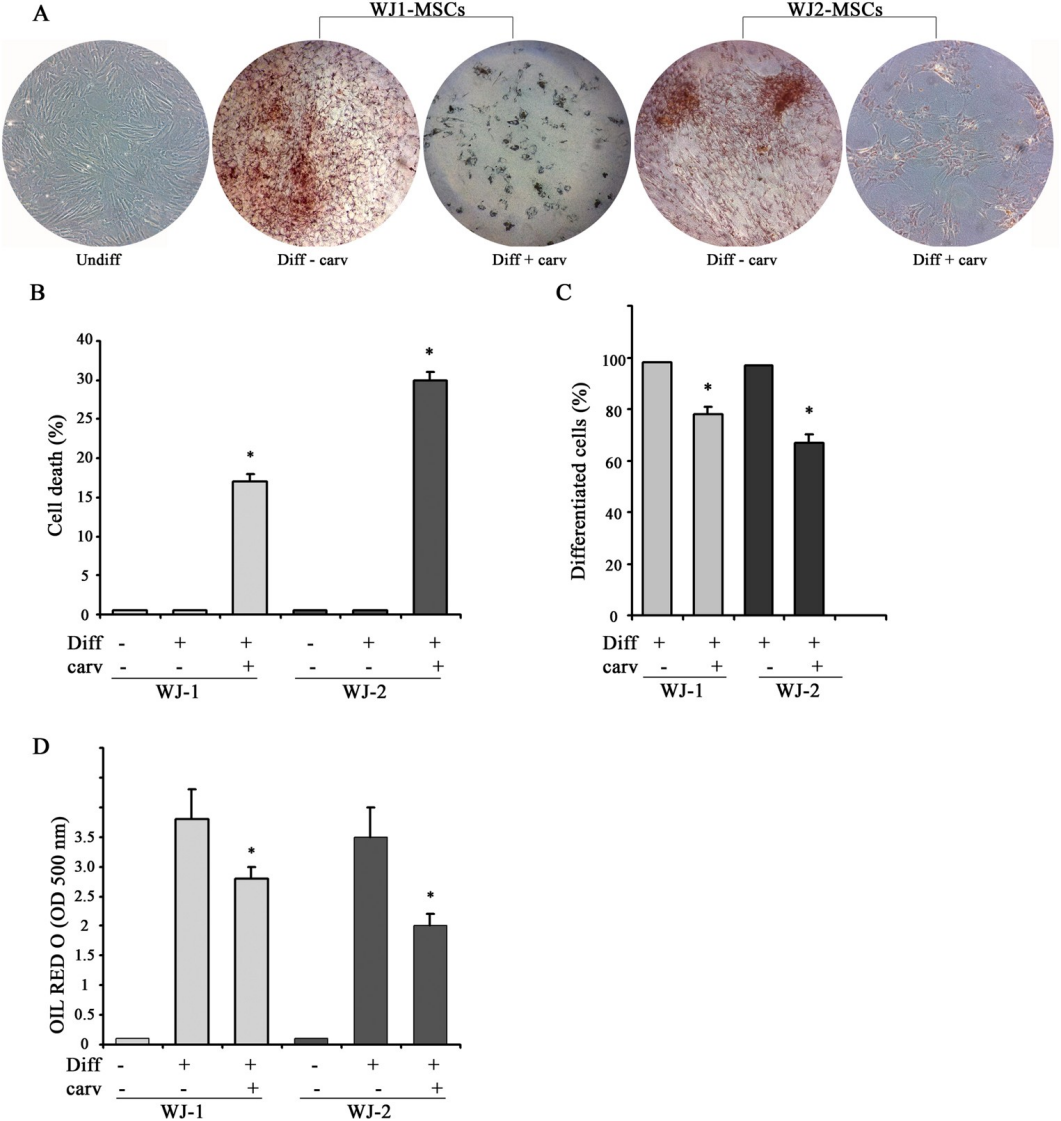
Reduction of adipocyte differentiation by carvacrol in WJ-MSCs cells. Two subcultures of Wharton’s jelly (WJ-MSCs), WJ-1 and WJ-2 cells, were grown in differentiation medium for 17 days (totally 80% differentiation) and treated with 25 μM carvacrol. (A) Triglyceride accumulation was visualized by Oil-Red O staining. Cells were observed at optical Zeiss microscope and photographed. (B) After 17 days of differentiation, cell viability was measured by Trypan blue staining; graph shows percentage of detached dead cells. (C) Four adjacent 1 mm squares were counted, blind to group, using an inverted microscope. The percentage of cells that underwent adipogenic differentiation was expressed as number of Oil-Red O positive/total cells. (D) Lipid accumulation was measured by using spectrophotometer. Data are presented as mean ± SD (*n 3)*. * p<0.05 (differentiated + carvacrol vs differentiated cells).

Having assessed that full adipogenic differentiation was achieved after 7 days in 3T3-L1 cells (90%) and after 17 days in WJ-MSCs cells (about 90%) (Figs 4A, 4B, 5A and 5C), adipogenic differentiation was induced in the presence or absence of carvacrol. As shown in Fig 4, carvacrol at 25 μM concentration reduced of about 40% cell differentiation in 3T3-L1 cell line, both after counting the Oil-Red O positive /total cells (Fig 4A and 4B) and after quantification of the Oil-Red O staining measured by spectrophotometer (Fig 4C). Of note, carvacrol at 25 μM concentration did not induce 3T3-L1 cell death, in fact trypan blue staining showed that cells undergoing adipogenic differentiation both in the presence and absence of carvacrol did not significantly change cell viability (data not shown).

Similarly, carvacrol (25 μM) reduced the WJ-1 and WJ-2 adipogenic differentiation, although to a lesser extent compared to 3T3-L1 cells. Thus, contrarily to what observed for 3T3-L1 cells, carvacrol induced cell death in both WJ-1 and WJ-2 cells (17% and 30%, respectively), during adipogenic differentiation, compared to differentiation alone and as measured by Trypan blue staining (Fig 5B). Concentration of carvacrol lesser than 25 μM, although reduced WJ1/2 cell death, did not significantly affect adipogenic differentiation in the remaining adherent cells (data not shown). Therefore, analysis of the remaining viable adherent cells showed that carvacrol reduced adipogenic differentiation of about of 22% and 27%, respectively for WJ-1 and WJ-2 cells, compared to the adherent differentiated cells without carvacrol (Fig 5D), both after counting the Oil-Red O positive/total cells (Fig 5C) and after quantification of the Oil-Red O staining by spectrophotometer (Fig 5D).

Altogether, these data indicate that carvacrol treatment was able to reduce adipogenic differentiation in both 3T3-L1 and WJ1/2 cells, although to a different extent, due to a slight toxic effect in WJ cells.

### Carvacrol reduces autophagy during adipogenic differentiation

Autophagy has been shown to regulate adipose mass and differentiation [30–35]. Inhibition of autophagy in pre-adipocytes reduces accumulation of TG and expression of transcription factors involved in adipocyte differentiation [29–34]. Therefore, we evaluate whether carvacrol could affect autophagy during adipogenic differentiation in 3T3 and WJ-1/2 cells.

**Fig 6:**
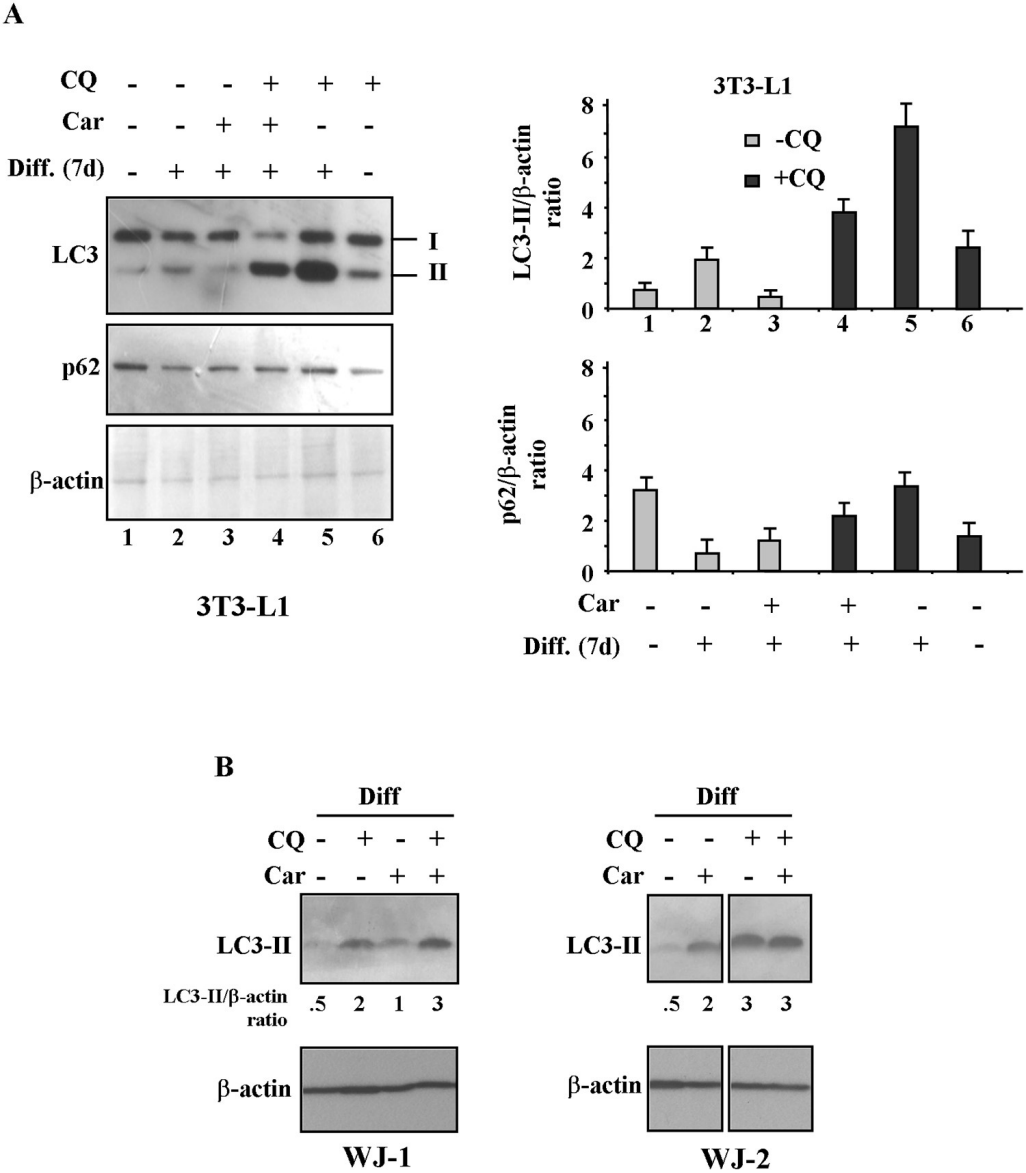
Effect of carvacrol on autophagy during adipogenic differentiation. Western blot and densitometric analysis of LC3 and p62 expression in 3T3-L1 (A) and WJ-1 and WJ-2 (B) cells undergoing adipogenic medium for 7 days (3T3-L1) and 17 days (WJ), respectively, with or without carvacrol treatment. Densitometric analysis of the 3T3-L1 signals is shown in the right panel. Bars depict means ± SE. (*n 2)*. * p<0.05. Lysosomal inhibitor Cloroquine (CQ) was added at 25μm for 4h before lysing cells for western blot analysis. Anti β-actin was used as protein loading control.

To this aim we evaluated the expression of microtubule-associated protein light chain 3 (LC3) protein that, after conversion from LC3-I to its autophagosome membrane-associated lipidated form LC3-II, is considered a cellular readout of autophagy [28]; to assess the autophagic flux, we used the chemical inhibitor of autophagic/lysosomal degradation chloroquine (CQ), as reported [28]. We found that the autophagic flux induced during differentiation in 3T3-L1 cells, as assessed by greater LC3-II expression (Fig 6A, lane 2 compared with lane 1), was reduced by carvacrol treatment (Fig 6A, lane 3 compared with lane 2). In agreement, reduction of p62 protein levels during adipogenic differentiation, confirming the degradation of the autophagic cargo (Fig 6A, lane 2 compared with lane 1), was in part inhibited by carvacrol treatment (Fig 6A, lane 3 compared with lane 2).

However, it cannot be excluded that p62 could be transcriptionally dowregulated by carvacrol.

The autophagic flux was evaluated by blocking the final step of autophagy (e.g., the autophagic/lysosomal degradation) by chloroquine (CQ) that allows to evaluate the autophagic flux by measuring the accumulation of LC3-II protein levels, as reported in the literature [28]. Fig 6 shows that CQ induced greater LC3-II accumulation in differentiated cells without carvacrol with respect to differentiated cells with carvacrol (Fig 6A, lane 5 compared with lane 4). These findings indicate that carvacrol is indeed impairing the autophagic flux during adipogenic differentiation in 3T3-L1 cells.

Of note, carvacrol did not seem to efficiently impair autophagy induced during adipogenic differentiation in WJ-1/2 cells, at least in terms of LC3-II levels (Fig 6B).

**Fig 7:**
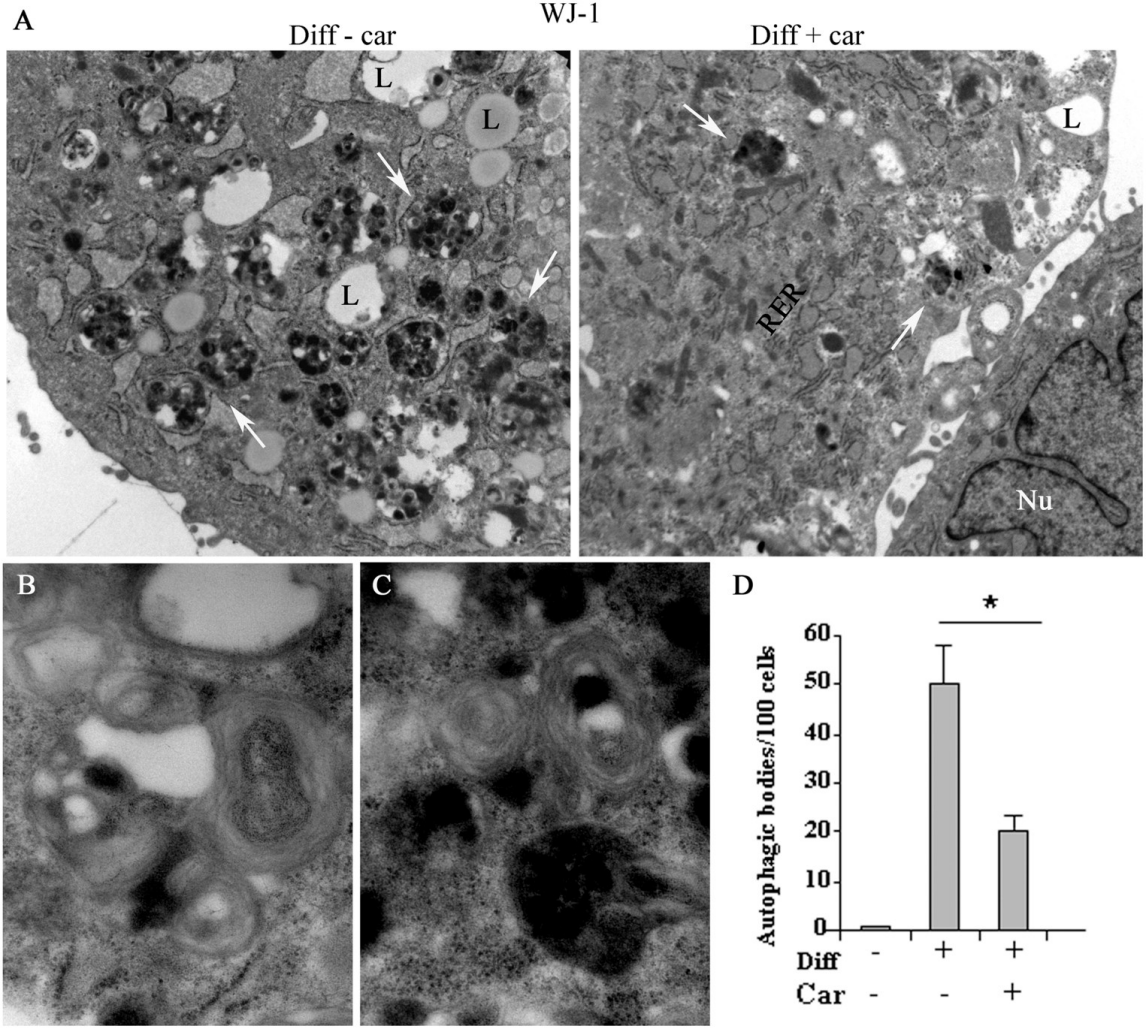
Transmission electron microscopy features of mature adipocyte. (A) Human WJ-1 cells cultured for 17 days in adipogenic differentiation medium without or with 25 μM carvacrol. In the left panel is shown the adipogenic differentiation with visible lipid vacuoles (L); in the right panel is shown the inhibition of adipogenic differentiation after carvacrol co-treatment with reduction of lipid vacuoles. The arrows indicate the autophagy bodies formed during adipogenic differentiation. Note that the formation of autophagy bodies was considerably reduced by carvacrol treatment. (B) (C) High magnification of autophagy bodies in Diff-carv sample. (D) Autophagic bodies were counted on 11000 magnification image and expressed as total x 100 cells. Bars depict means ± SD. (*n 3)*. * p<0.05. Nu:nucleus, cy:cytoplasm, L: lipid vacuoles. RER: rough endoplasmic reticulum. Original magnification: (A), x5600; (B) (C) x44000.

However, ultrastructural analysis by TEM revealed that the formation of autophagic bodies during adipogenic differentiation in WJ-1 cells was reduced by carvacrol treatment (Fig 7A and 7D), indicating that carvacrol was affecting autophagy also in WJ cells.

### Effect of carvacrol on ChREBP activity during adipogenic differentiation

**Fig 8:**
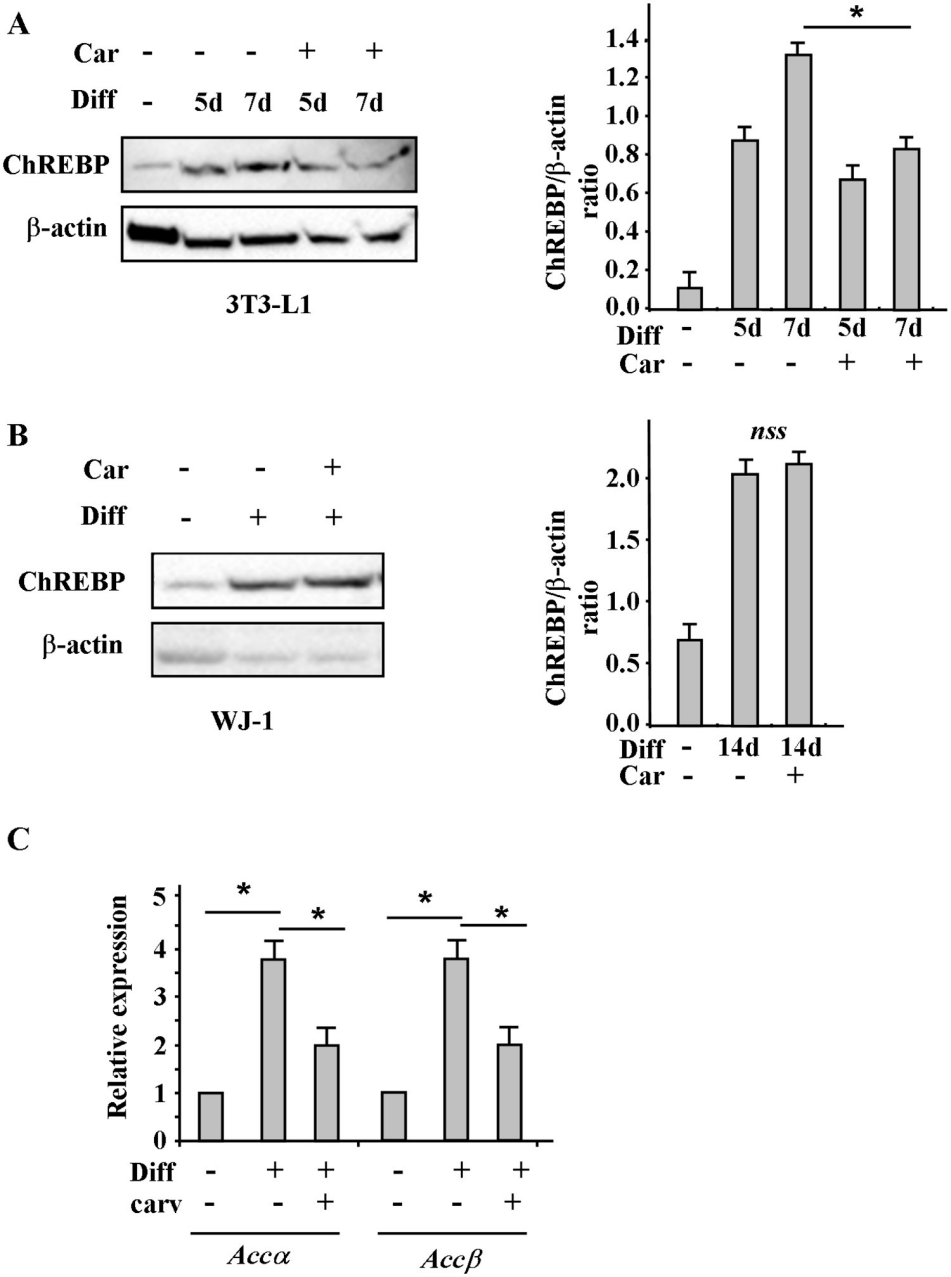
Effect of carvacrol on ChREBP acrivity during adipogenic differentiation. Western blot and densitometric analysis of ChREBP in 3T3-L1 (A) and WJ-1 (B) cells undergoing adipogenic differentiation for, respectively, 5 to 7 days and 17 days with or without carvacrol co-treatment. Anti β-actin was used as protein loading control. (C) Expression of ChREBP target genes encoding enzymes involved in lipogenesis in WJ-1 cells treated as in (B). ChREBP target genes were detected by RT-PCR analysis and gene expression, measured by densitometry, was normalized to 28S levels, ±SD and plotted as relative mRNA expression. *p< 0.05 (diff compared to undiff; and diff+carv compared to diff). nss = not statistically significant.

Finally, we analysed if carvacrol might modulate ChREBP since it is usually activated during adipogenic differentiation. Western blot analysis showed that the ChREBP protein levels, increased in 3T3-L1 cells during adipogenic differentiation, were reduced by carvacrol treatment (Fig 8A), as confirmed by densitometric analysis (Fig 8A). On the other hand, ChREBP levels slightly increased in WJ-1 cells during adipogenic differentiation but were not efficiently modified by carvacrol treatment (Fig 8B). However, the ChREBP-induced adipogenic target genes, that is Acetyl-CoA carboxylase (ACC) α and β, were induced during adipogenic differentiation and reduced by carvacrol treatment (Fig 8C), indicating an effect of carvacrol on ChREBP activity.

Furthermore, ultrastructural immunolocalization of ChREBP protein was determined in WJ-1 cells by using the TEM immunoelectron microscopy gold technique. The use of bio-acrylic resin allowed to preserve the protein antigenicity. For each sample at least 100 cells were examined counting the total number of gold particles per unit areas, in the cytoplasm and nucleus (10 cm^2^ at 11000 of magnification) (Table 2).

**Table 2:** Immunogold quantitative evaluation of ChREBP protein in cytoplasmic and nuclear compartments of undifferentiated, differentiated and carvacrol-differentiated cells.

	Undifferentiated	Differentiated	Differentiated +Carvacrol
Nucleus	38.5 ± 3.1	42 ± 5.2	42 ± 2.8
Cytoplasm	69.45 ± 5.9	101.2± 9.1	97 ± 8.7 *

Number of particles per 10 cm^2^ at x 11000

Data are the mean of at least five observations. ± SEM.

* p<0.05 vs (differentiated+carvacrol *vs* differentiated)

**Fig 9:**
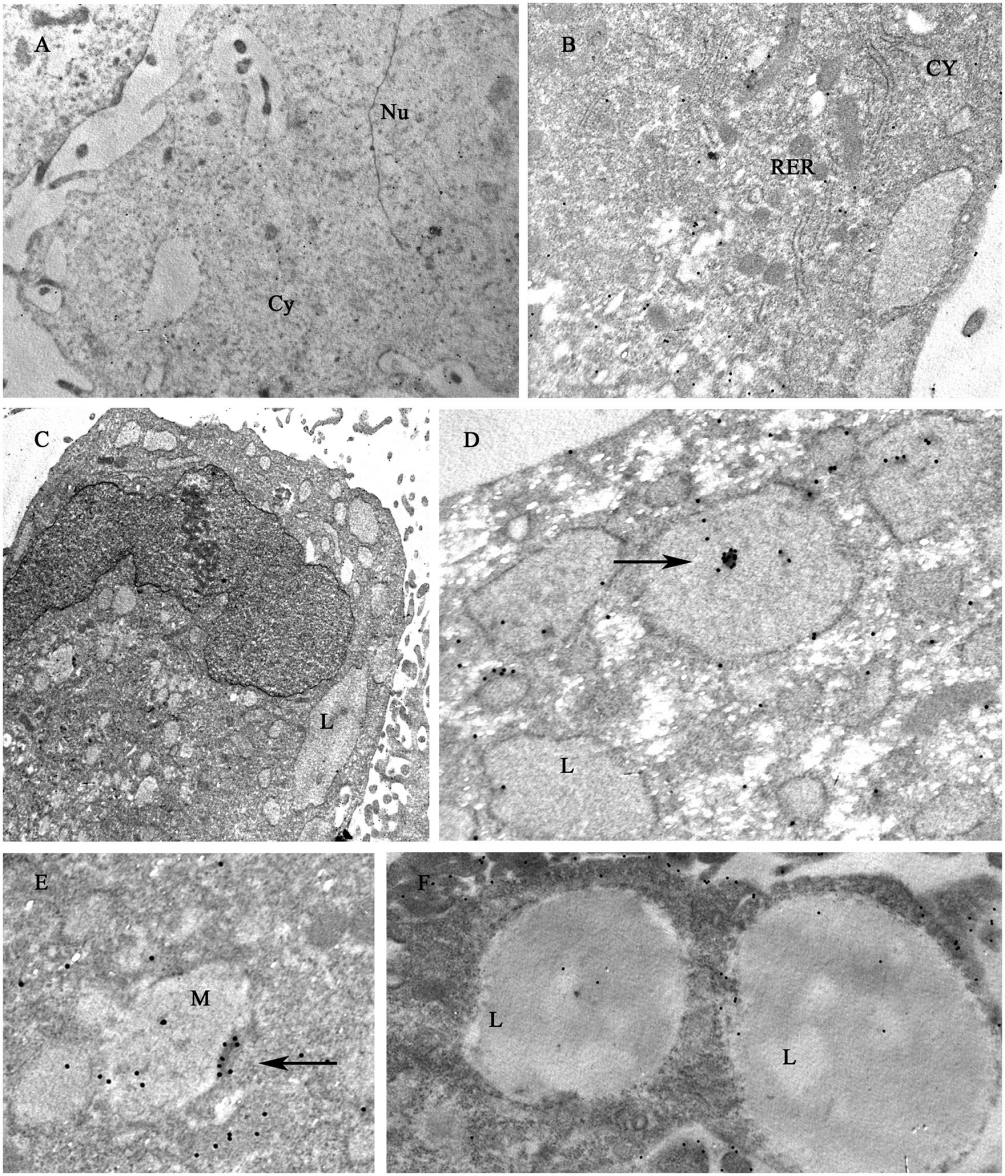
Immunogold detection of ChREBP protein in human WJ-MSCs cells. Electron micrographs of uncounterstained sections of human WJ-1 cells cultured for 17 days in adipogenic differentiation medium with or without 25 μM carvacrol. (A) Undifferentiated: 20nm gold particles are distributed in nuclear and cytoplasmic compartments. (B) Differentiated cells in presence of carvacrol: cells are lower differentiated, 20-nmgold granules are distributed in nuclear and cytoplasmic compartments, inside the rough endoplasmic reticulum (RER). (C) Differentiated cells without carvacrol (control). (D) (E) (F) higher magnification of (C). The gold particles detected showed a predominant localization in the rough endoplasmic reticulum (C) mitochondria (E), lipid vacuoles (D) (F). Squares (10 cm^2^ at x 11000) were randomly chosen to count the number of 20-nm gold particles in the various cell compartments. Nu: nucleus, cy: cytoplasm, L: lipid vacuoles. RER: rough endoplasmic reticulum. M: mitochondrion. Arrows indicate gold clusters Original magnification: (A),(B),(C) x11000; (D),(E),(F) x22000.

As shown in Fig 9A, the immunolabeling was detectable both in nuclear and cytoplasmic compartment of undifferentiated cells. Interestingly, after 17 days of differentiation, the cytoplasmic labeling was increased by more than 30% compared with undifferentiated cells (Table 2, Fig 9C), whereas the nucleus labeling remained the same. The gold particles detected in differentiated WJ-1, showed a predominant localization in the rough endoplasmic reticulum (Fig 9B), mitochondria (Fig 9E) and lipid vacuoles (Fig 9D–9F). ChREBP was less expressed in cytoplasmic compartment of cells differentiated in presence of carvacrol (Fig 9B).

## Discussion and conclusions

With the worldwide prevalence of obesity and its clinical implications new anti-obesity drugs are urgently needed. Many natural products have been used since ancient times for their benefits and some of them have recently been *re-discovered* for their anti-adipogenic effect.

Energy homeostasis is a biological process that regulates the balance between energy inflow (food intake) and energy expenditure. When the two processes are imbalanced obesity may develop. Obesity is a result of accumulation of white adipose tissue where excess of food energy is stored to form triglycerides (TG). The process of the production of new fat cells is known as adipogenesis. Many natural products inhibit adipogenesis including flavonoids and antocyanins [48, 51] and among them the antiadipogenic effect of carvacrol has been previously demonstrated [45]. Here we found that carvacrol reduced adipogenic differentiation in both murine 3T3-L1 cells (Fig 4A–4C), and human Wharton’s jelly derived mesenchymal stem cells (WJ-MSCs) (Fig 5A, 5C and 5D), and that such reduction correlated with reduction of autophagy and ChREBP activity, although in a different extent between cells.

**Fig 10:**
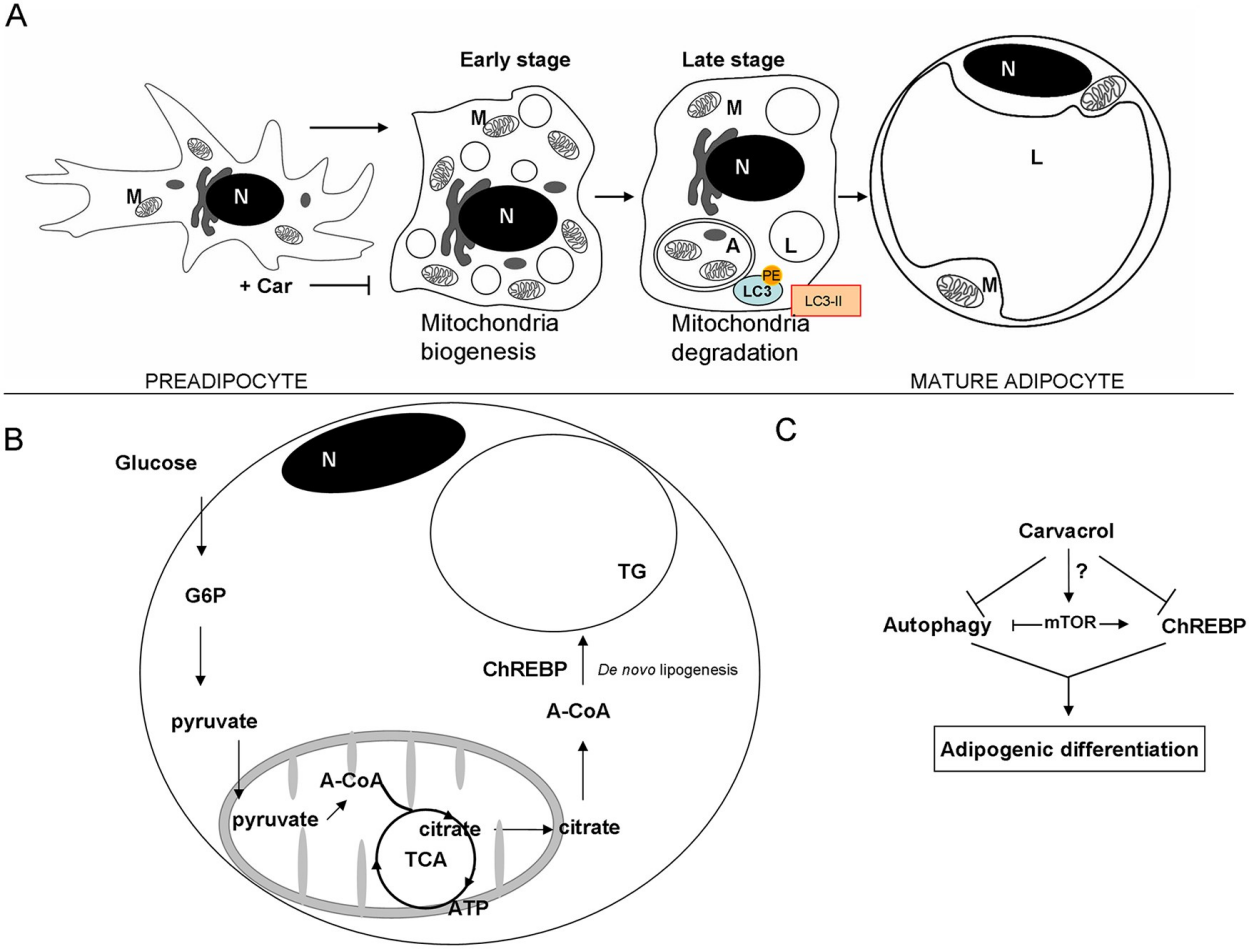
Schematic model showing the hypothesis of how carvacrol reduced adipogenesis. Schematic representation showing the (A) formation of the mature adipocyte during adipogenic differentiation and the role of autophagy; (B) the role of glucose and ChREBP during TG formation; and (C) how carvacrol might reduce adipogenic differentiation. N: nucleus; A:autophagosome; L:lipid droplet; M:mitochondrion.

The adipocyte differentiation and adipogenesis are a multistage process including commitment of mesenchymal stem cells to a preadipocyte fate, an early stage of differentiation and the terminal differentiation. During early stage, to form the mature adipocyte with a different cellular structure, the cells change their subcellular morphology: increase in mitochondrial biogenesis, in cytoplasmic volume and with formation of lipid droplets. In the late state of differentiation, different cellular rearrangements are necessary, especially cytoplasmic, with degradation and removal of the mitochondria [52]. During this process, autophagy and mitophagy (a mitochondria specialized type of autophagy) play an important role in cytoplasm remodelling (Fig 10A). Autophagy plays an important role in the elimination of mitochondria. Indeed, knock-out mice for autophagy related genes have anti-obesity and anti-diabetic phenotype [30]. The autophagy mechanism (macro-autophagy) initiates with the formation of the phagophore assembly followed by the autophagosome formation. Phagophore membrane elongation and autophagosome completion require two ubiquitin-like conjugation pathways. The first produces the ATG5–ATG12 conjugate, whereas the second results in the conjugation of phosphatidylethanolamine (PE) to LC3 (the microtubule-associated protein 1 light chain 3). PE-conjugated LC3 (LC3-I) is required for the expansion of the autophagic membranes and for its ability to recognize autophagic bodies and the fusion of autophagosomes with lysosomes. The conversion of LC3 to LC3-I and then to LC3-II is correlated with the assembly of the membrane of autophagosome. LC3 attached to the autophagosome membrane binds p62, which in turn binds ubiquitinated substrate proteins [53]. Phosphorylation of p62 mainly occurs on selective cytotoxic autophagic cargos. Thus the p62 protein recognizes toxic cellular waste, which is then scavenged by the autophagic process [54]. Lack of autophagy leads to accumulation of p62, which is toxic for liver cells and induces a cellular stress response. The multifunctional adaptor protein p62/sequestome1 was the first described selective autophagy receptor [55]. Knockout of autophagy genes leads to abnormal p62 aggregate accumulation in several cell types [56]. The autophagic adaptor p62/SQSTM1 is essential for the clearance of mitochondria [52]. For these reasons, both p62/SQSTM1 and LC3, in its isoforms I and II, are well established autophagy markers, considering, however, that p62 change is cell type specific and there is not a clear correlation between increase in LC3-II and decrease of p62. In fact, in some cell types, there is no change in the overall amount of SQSTM1 despite strong levels of autophagy induction. In agreement, we found that carvacrol considerably reduced the autophagy marker LC3-II, induced by adipogenic differentiation, in 3T3-L1 (Fig 6); similarly, TEM showed that autophagic bodies increased during adipogenic differentiation in WJ-1/2 cells were strongly reduced after carvacrol treatment, indicating that carvacrol might reduce adipogenic differentiation in part by modulating autophagy (Fig 7). Furthermore to evaluate and measure autophagy activity, through the chemical inhibitor of autophagic/lysosomal degradation chloroquine (CQ), we found that the autophagic flux induced during differentiation in 3T3-L1 cells, as evidenced by LC3-II accumulation, was reduced by carvacrol treatment, (Fig 6A).

De novo lipogenesis process is driven to some extent by ChREBP [20] that is activated by increased insulin signalling in response to high glucose level [21, 22] (Fig 10B). ChREBP specifically binds the carbohydrate response element (Chre) of the lipogenic genes Acetyl CoA carboxylase, Fatty acid synthase and Pyruvate kinase (LPK) [23]. In agreement, we showed that carvacrol reduced the ChREBP expression and ACCα and β expression, activity during adipogenic differentiation. The increase in ChREBP in the initial differentiation phase is probably correlated to the early autophagic stage, with increase of mitochondrial biogenesis and elongation, higher availability of ATP and Acetyl-CoA, necessary for de novo lipogenesis [23]. These elongated mitochondria may possess more cristae and generate more ATP, energy requirement for DNL. It is established that the kinase mTOR exerts its functions by entering into two complexes, termed mTORC1 and mTORC2 [57], and the mTOR complex 2 (mTORC2) promotes expression of the lipogenic transcription factor ChREBP, and de novo lipogenesis, by controlling in part glucose uptake into adipocytes, independently of the classic AKT pathway [58]. Therefore, since autophagy is commonly regulated by the kinase mTOR [56] and mTORC2 promotes expression of ChREBP, next studies will be needed to evaluate if carvacrol might modulate mTORC2 complex during adipogenic differentiation (Fig 10C).
